# Supplementary material for: Reconciling Mining with the Conservation of Cave Biodiversity: A Quantitative Baseline to Help Establish Conservation Priorities
Source: PLoS One. 2016 Dec 20;11(12):e0168348. doi: 10.1371/journal.pone.0168348 (PMC5173368; doi:10.1371/journal.pone.0168348)
Supplement: S1 Dataset — (ZIP) [file pone.0168348.s002.zip › Taxa/Serra Norte/SN_2013/N4E/Lista N4E-39.pdf]

## CAVIDADE N4E-0039

| Classe | Ordem | Fam/Outros | Gên/Outros | Espécie | S-U | U-S | Zon. |
|---|---|---|---|---|---|---|---|
| Arachnida | Acari | Laelapidae | | sp.3 | | X | P |
| Arachnida | Amblypygi | Phrynidae | *Heterophrynus* | *longicornis* | 3 | 4 | E/P |
| Arachnida | Araneae | Barychelidae | | jovem | | 2 | E |
| Arachnida | Araneae | Caponiidae | | jovem | | X | P |
| Arachnida | Araneae | Corinnidae | | jovem | 1 | 2 | E/P |
| Arachnida | Araneae | Ctenidae | | jovem | | 1 | E |
| Arachnida | Araneae | Drymusidae | *Drymusa* | *spelunca* | | X | E |
| Arachnida | Araneae | Ochyroceratidae | *Ochyrocera* | sp.1 | X | X | E/P |
| Arachnida | Araneae | Ochyroceratidae | *Speocera* | sp.1 | X | X | P |
| Arachnida | Araneae | Pholcidae | *Mesabolivar* | sp.1 | X | X | E |
| Arachnida | Araneae | Salticidae | | jovem | | X | E |
| Arachnida | Araneae | Theridiidae | *Theridion* | sp.1 | X | X | E |
| Arachnida | Araneae | Theridiosomatidae | *Ogulnius* | sp.1 | X | | P |
| Arachnida | Araneae | Theridiosomatidae | *Plato* | sp.1 | X | | E/P |
| Arachnida | Opiliones | Escadabiidae | | sp.1 | | X | P |
| Arachnida | Opiliones | Escadabiidae | | sp.2 | X | | P |
| Arachnida | Opiliones | Stygnidae | *Protimesius* | *gracilis* | X | | P |
| Arachnida | Pseudoscorpiones | Chernetidae | *Spelaeochernes* | sp.1 | | X | P |
| Chilopoda | Scolopendromorpha | Scolopocryptopidae | *Newportia* | sp.1 | 2 | | P |
| Diplopoda | Polydesmida | Chelodesmidae | | sp.3 | | 2 | P |
| Diplopoda | Polydesmida | Fuhrmannodesmidae | | sp.1 | | X | P |
| Diplopoda | Polydesmida | Pyrgodesmidae | | sp.1 | | X | P |
| Diplopoda | Spirostreptida | Pseudonannolenidae | *Pseudonannolene* | jovem | 4 | 3 | E/P |
| Entognatha | Collembola | Isotomidae | | sp.1 | X | | P |
| Entognatha | Collembola | Isotomidae | | sp.2 | | X | P |
| Entognatha | Collembola | Paronellidae | | sp.4 | | X | E/P |
| Entognatha | Collembola | Sminthuroidea | | sp.2 | X | X | P |
| Entognatha | Diplura | Campodeidae | | sp.1 | X | X | E/P |
| Insecta | Blattodea | Blaberidae | | jovem | 2 | | P |
| Insecta | Coleoptera | Carabidae | *Coarazuphium* | sp.n.1 | X | | P |
| Insecta | Coleoptera | Curculionidae | Scolytinae | sp.4 | | X | P |
| Insecta | Coleoptera | Staphylinidae | Pselaphinae | sp.20 | X | | E |
| Insecta | Diptera | Dolichopodidae | | sp. | X | | E |
| Insecta | Diptera | Psychodidae | Phlebotominae | sp. | X | X | E/P |
| Insecta | Heteroptera | Cydnidae | | jovem | | X | P |
| Insecta | Heteroptera | Reduviidae | Emesinae | sp. | | 1 | E |
| Insecta | Homoptera | Cixiidae | | jovem | X | X | E/P |
| Insecta | Hymenoptera | Formicidae | *Solenopsis* | sp.1 | X | | P |
| Insecta | Hymenoptera | Formicidae | *Camponotus* | sp.1 | X | X | E/P |
| Insecta | Hymenoptera | Formicidae | *Eurhopalothrix* | sp.1 | | X | P |
| Insecta | Hymenoptera | Formicidae | *Hypoponera* | sp.1 | X | X | E/P |
| Insecta | Hymenoptera | Formicidae | *Pachycondyla* | sp.2 | X | X | E/P |
| Insecta | Isoptera | Termitidae | *Nasutitermes* | sp. | X | X | E |
| Insecta | Isoptera | Termitidae | *Termes* | sp. | | X | E |
| Insecta | Lepidoptera | | | jovem | | X | E |
| Insecta | Orthoptera | Gryllidae | Nemobiinae | sp. | | 1 | P |
| Insecta | Orthoptera | Phalangopsidae | *Paraclodes* | jovem | 10 | | E/P |
| Insecta | Orthoptera | Phalangopsidae | *Phalangopsis* | jovem | 67 | 15 | E/P |
| Insecta | Psocoptera | Epipsocidae | *Epipsocus* | sp.2 | X | | P |

| | | | | | | | |
|---|---|---|---|---|---|---|---|
| Gastropoda | Pulmonata | Bulimulidae | *Naesiotus* | sp. | X | X | P |
| Gastropoda | Pulmonata | Subulinidae | | sp.5 | | X | P |
| Gastropoda | Pulmonata | Systrophiidae | *Happia* | sp. | | X | P |
| Malacostraca | Isopoda | Scleropactidae | *Circoniscus* | *buckupi* | | X | P |
| Symphyla | | Scutigerellidae | | sp. | X | X | P |
| Amphibia | Anura | Strabomantidae | *Pristimantis* | cf. *fenestratus* | 8 | 3 | E |
| Mammalia | Chiroptera | Emballonuridae | *Peropteryx* | *kappleri* | 1 | 3 | P |
| Mammalia | Chiroptera | Phyllostomidae | *Carollia* | *perspicillata* | 2 | 1 | P |
| Mammalia | Rodentia | Cricetidae | *Rhipidomys* | sp. | | 1 | P |
